# Microvascular and Structural Characterization of Birdshot Chorioretinitis in Active and Inactive Phases

**DOI:** 10.3390/biomedicines12102414

**Published:** 2024-10-21

**Authors:** Aina Moll-Udina, Marina Dotti-Boada, Anabel Rodríguez, Maite Sainz-de-la-Maza, Alfredo Adán, Victor Llorenç

**Affiliations:** 1 Clinic Institute of Ophthalmology (ICOF), Clínic Hospital of Barcelona, 08028 Barcelona, Spain; dotti@clinic.cat (M.D.-B.); anrodrig1@clinic.cat (A.R.); msainz@clinic.cat (M.S.-d.-l.-M.); amadan@clinic.cat (A.A.); vllorens@clinic.cat (V.L.); 2 August Pi i Sunyer Biomedical Research Institute (IDIBAPS), 08036 Barcelona, Spain

**Keywords:** birdshot, biomarkers, chorioretinitis, imaging, non-infectious uveitis, ocular inflammation, optical coherence tomography angiography, quiescence, uveitis

## Abstract

**Objective:** This study aimed to examine microvascular changes and identify predictors of short-term quiescence in active birdshot chorioretinitis (BSCR). **Methods:** An observational, prospective, 12-month follow-up cohort study was conducted. BSCR eyes were clinically assessed at baseline, categorized as active or inactive, and reevaluated at 12 months. Based on their clinical activity at both timepoints, eyes were divided into three subgroups: active-to-inactive (A-I), consistently active (A-A), and consistently inactive (I-I). Structural OCT, OCT-angiography (OCT-A), and ultra-widefield imaging were utilized. Exam data from fundus and nasal subfields were analyzed for microvascular changes and quiescence predictors. **Results:** Sixty eyes from 30 BSCR patients (47% women, 53% men, mean age 59.7 ± 12.3 years) were included. In the A-I group (16 eyes), vascular density and perfusion indices increased in all subfields post-quiescence, contrasting with the other groups. Perifoveal looping in the superficial capillary plexus predicted quiescence at 12 months compared with the A-A group. **Conclusions:** Vascular density rises after complete inflammation control in BSCR, and perifoveal capillary loops serve as potential predictors of short-term quiescence in active BSCR.

## 1. Introduction

Birdshot chorioretinitis (BSCR), a rare form of posterior uveitis, accounts for 5 to 8% of all posterior uveitis cases. Primarily affecting intraocular tissues, particularly chorioretinal structures, this condition is prevalent in middle-aged Caucasians [1,2]. The precise pathophysiology remains elusive; however, it is widely hypothesized to be an immune-mediated disorder closely associated with the human leukocyte antigen (HLA) specific allele HLA-A29 [3,4]. Therefore, some authors advocate for its designation as “HLA-A29 BSCR”, as the presence of HLA-A29 is considered a diagnostic prerequisite [5].

BSCR is a bilateral, sometimes asymmetrical eye condition characterized by oval, creamy yellow lesions at the back of the eye, particularly in the inferonasal arcade around the optic nerve—hence its name [6]. In active cases, angiography typically reveals extended inflammation of retinal capillaries, with dark spots on fluorescein and indocyanine green exams [7]. However, our understanding of capillary abnormalities, vascular density, and blood flow changes in the superficial retinal capillary network during BSCR’s course is limited, especially in nasal fields, where significant tissue changes may occur later on.

There are various BSCR phenotypes, ranging from benign, with a favorable prognosis not requiring treatment, to a progressive form that, despite treatment, leads to severe chorioretinal atrophy [2,8]. Given its slow and progressive nature, diligent and thorough monitoring is crucial for effective treatment adjustment.

Traditionally, BSCR monitoring has relied on visual fields and electroretinograms [9,10,11], which are valuable for tracking long-term functional progression when structural damage is well-established and largely irreversible. Recent advances in multimodal imaging help explore potential biomarkers that may predict short-term treatment response and monitor inflammation activity.

In BSCR, inflammatory involvement of the choroid and the retina might be independent. The choroidal findings are well described, and they are clinically more apparent in the nasal fields. Whether underlying choroidal inflammation enhances retinal microvascular involvement or not is not clear. However, being a diffuse retinal capillaritis a distinctive finding in BCR, microvascular pathophysiological changes are poorly understood [12]. Moreover, the retinal inflammatory damage is probably the main cause of these patients’ visual and functional impairment, secondary to diffuse retinal atrophy [2].

Optical coherence tomography angiography (OCT-A) represents a recent addition to the armamentarium for investigating microvascular retinal networks. Together with high-resolution en-face retinal color OCT maps and ultra-widefield (UWF^®^) imaging, these tools hold promise as potential sources of new markers to enhance our understanding of the pathophysiological changes in BSCR [13].

This study seeks to scrutinize microvascular and structural changes in active and inactive BSCR stages, aiming to identify potential imaging markers that can predict short-term outcomes.

## 2. Materials and Methods

A prospective observational study was conducted at the Clínic Hospital of Barcelona, Spain, a tertiary referral center. Patient enrollment occurred consecutively from December 2019 to July 2022, with a 12-month follow-up period. The study protocol received approval from the Clinic Hospital’s Ethics Committee (HCB/2020/0472), adhering to principles outlined in the Declaration of Helsinki (October 2013). All participating patients provided written informed consent.

Inclusion criteria encompassed a BSCR diagnosis according to Levinson’s criteria or the SUN group classification [7,14]. Exclusion criteria involved the presence of visual axis opacities hindering accurate fundus visualization, suboptimal imaging quality on OCT or OCT-A exams (Q < 7/10), or the coexistence of concurrent chorioretinal comorbidities. There were no significant differences in vitreous haze or anterior chamber cell scores among groups that could jeopardize the quality of the images or bias the observed trends in vascular density and perfusion index.

Treatments before and after inclusion followed general practice guidelines for chronic bilateral posterior uveitis: patients with active disease received systemic immunomodulation with an anti-metabolite drug. Adalimumab or tocilizumab were added or used as monotherapy, in cases of persistent activity or intolerance, respectively. Local dexamethasone implants were used as adjuncts in refractory cases, especially those with unilateral reluctant macular edema.

The protocol was implemented at two timepoints: baseline and 12 months, and comprised the following steps: 1. Double clinical assessment: Conducted by two independent senior uveitis specialists (A.A., M.S.), including best-corrected visual acuity (Snellen converted to logMAR), tonometry (Goldmann), biomicroscopy (grading anterior chamber cells according to the Standardization of Uveitis Nomenclature), and indirect funduscopy (vitreous haze score); 2. OCT: Utilizing spectral-domain OCT (Cirrus HD-OCT^®^, Carl Zeiss Meditec, Dublin, CA, USA) with a macular cube of 512 × 128 μm within a 6 × 6 mm area to automatically obtain central macular thickness (CMT) and macular volume (MV); 3. UWF^®^ pseudocolor retinography (Optos PLC, Dunfermline, UK): recorded number, size, location, and pigmentation of BSCR spots; 4. UWF^®^ fluorescein angiography (FA): Analyzed according to the Angiography Scoring for Uveitis Working Group (ASUWOG) [15] with a cutoff point of >5 points to determine activity; 5. UWF^®^ Indocyanine Green Angiography (ICG): Performed at the investigator’s discretion in selected cases due to healthcare authorities’ restrictions for human use; 6. Blind classification of eyes: BSCR eyes were blindly categorized as “active” or “inactive” by experts (A.A. and M.S.), with any discrepancies resolved by a third expert (V.L.). Activity was defined if anterior chamber cells (ACC) ≥ 1+, vitreous haze (VH) ≥ 1+, ASUWOG ≥ 5, with or without CMT > 300 µM. Inactivity was defined as ACC ≤ 0.5+, VH ≤ 0.5+, and ASUWOG < 5. The presence of clearly defined rice-shaped hypofluorescent dark dots on ICG late frames supported the active status in those eyes that were deemed doubtful by other tests and clinical exams.

The following tests were performed as per the protocol: 6.1. OCT cube acquisition protocol of the inferonasal areas to automatically quantify nasal retinal thickness (NRT) and nasal volume (NV); 6.2. Enhanced deep imaging (EDI) OCT recording choroidal thickness and the integrity of the ellipsoid layer (normal, damaged, or absent) in the macular and inferonasal area. For nasal captures, the ellipsoid zone and choroidal thickness were evaluated, 1 mm apart from the optic disc edge at V o’clock, using a second-order nasal vessel as a reference for both timepoint captures; 6.3. OCT-A: Angioplex^®^ (Carl Zeiss Meditec, CA, USA) 6 × 6 mm frame, en-face thickness maps of the fundus field were exported in a color scale. The averaged index of the amount of red and blue in RGB (Red-Green-Blue) units per area in pixels was calculated. Averaged thickened retinal index (ATRI) and averaged atrophy retinal index (AARI) were studied following thickness map processing, as described previously [16]; 6.4. OCT-A 3 × 3 mm and 6 × 6 mm Retinal Vasculature Analysis: Retinal vascular density (VD) and perfusion index (PI) were automatically determined by the Angioplex^®^ interface using an ETDRS grid in the superficial capillary plexus (SCP) of the fundus, superonasal, and inferonasal fields. To allow reproducible identification of extramacular image location (nasal subfields), an area closely adjacent to and inferior to the optic disc was chosen.

Additionally, the area, perimeter, and circularity of the foveal avascular zone (FAZ), along with qualitative capillary assessments (telangiectasias, increased intercapillary spaces, capillary irregularity, and capillary loops) of the SCP were studied using a 3 × 3 mm and 6 × 6 mm Angioplex^®^ frame in the fundus field.

Classification into subgroups was finally determined by considering initial and final activity classifications. Three primary subgroups were established: A-I (active-to-inactive)—Eyes initially classified as active that transitioned to an inactive state; A-A (active-to-active):—Eyes that retained an active classification throughout both the initial and final assessments; and I-I (inactive-to-inactive):—Eyes that maintained an inactive classification throughout the study. It is noteworthy that a subgroup consisting of inactive eyes that reactivated (I-A) was intentionally excluded from analysis due to its limited size (2 eyes), which lacked statistical power for meaningful interpretation. This classification methodology provides a comprehensive framework for understanding the dynamic changes in eye activity throughout the study.

### Statistical Analyses

Categorical variables are presented as absolute numbers and percentages, while quantitative variables are expressed as the mean and standard deviation or the median and interquartile range. The three-category (A-A, A-I, I-I) polychotomous variable “outcome group” was characterized by comparing the mean values of each category to the mean of the entire population. Quantitative variables characterizing the “outcome group” were compared using the Mann–Whitney U or Kruskal–Wallis test, depending on the number of categories. Qualitative variables characterizing the “outcome group” were assessed with an independence test (Fisher’s exact or Chi-square independence test, depending on normality). Bonferroni’s and Yates’ continuity corrections were used in multiple comparison analysis. To investigate potential independent outcome predictors, considering high collinearity among covariates, a partial least squares discriminant analysis (PLS-DA) was implemented between groups A-A and A-I. PLS-DA combines principal components analysis and logistic regression. Covariates with *p* ≤ 0.1 for univariate analysis or those deemed clinically meaningful (age, evolution time to baseline, sex) were included in the model. A significance level of less than 5% in the alpha error was established as statistically significant.

## 3. Results

Thirty-two HLA-A29-positive Caucasian patients were enrolled, accounting for a total of 64 eyes. In thirteen patients, one eye was assigned to a different outcome group than the fellow. Four eyes from two patients were excluded due to concurrent chorioretinal comorbidities—one patient with myopia magna and the other with a macular neovascular membrane in one eye and macular scarring in the other. Consequently, 60 eyes from 30 patients were ultimately included for analysis (47% women, 53% men, mean age 59.7 ± 12.3 years). Agreement in classification (active/inactive) was reached for 56 of the 60 included eyes (93%). Four eyes (corresponding to three patients) required a third-party evaluation and classification. Causes of disagreement were a few fuzzy remaining hypofluorescent dark dots in one eye in the ICG exam at 12 months, classified finally as inactive; diffuse intense capillaritis on FA in two eyes, classified finally as active at baseline and 12 months; and one eye with chronic mild macular edema and epiretinal membrane at baseline that was classified as inactive after a third expert evaluation.

Among the enrolled patients, twenty-nine (97%) had HLA-A29:02 haplotypes and one the A29:01 haplotype. Twenty-eight eyes (46.66%) belonged to female patients, with an overall mean age of 59.7 ± 12.3 years and a mean evolution time until inclusion of 111 ± 67.9 months.

### 3.1. Active-Inactive Subgroup

**Figure 1:**
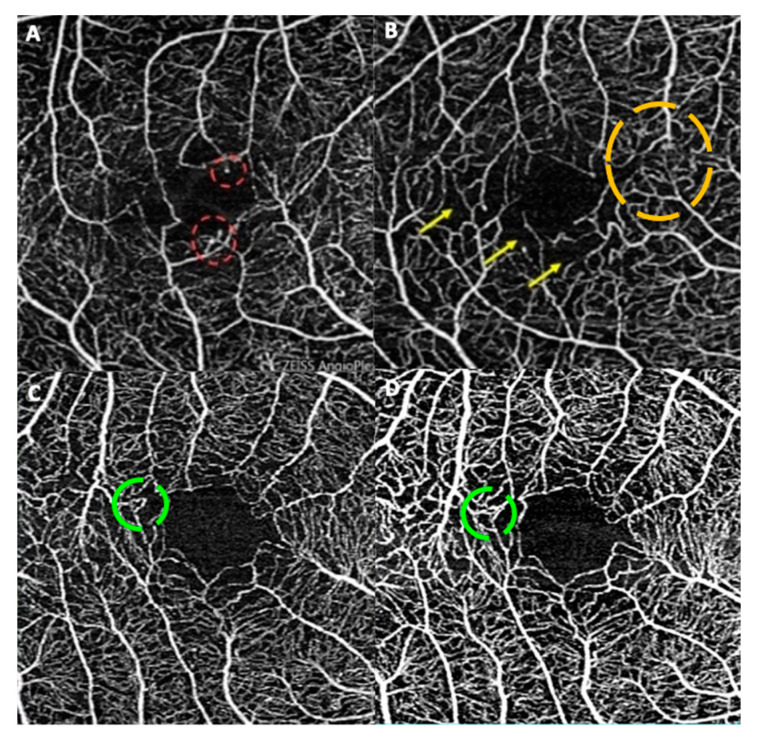
Qualitative capillary abnormalities at the superficial capillary plexus of 3 × 3 OCT-A. Telangiectasias, seen as capillary dilatations (dashed red circle), are visualized in (**A**). There are greatly increased intercapillary spaces, especially perifoveal in (**B**) (yellow arrow) and capillary irregularities (dashed orange circle). A perifoveal capillary loop (dashed green circle in (**C**)) at baseline that faded at 12 months (**D**) is seen in an eye from the active-inactive group.

**Figure 2:**
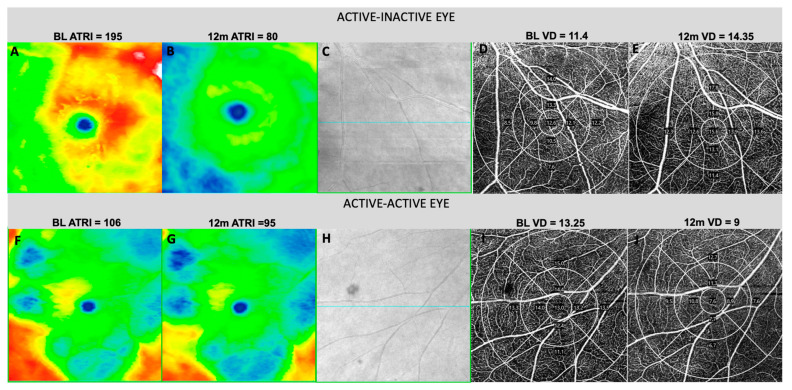
Top Row—Transition from active to quiescent BSCR eye from baseline to 12 months. En-face all-retina thickness color-scaled map: A notable reduction in averaged thickened retinal index (ATRI) is observed (**A**,**B**), and an increase in total vascular density (VD) at the inferonasal subfield (**D**,**E**) is seen in the superficial capillary plexus (SCP) of 6 × 6 OCT-A. Bottom Row—Persistence of activity in BSCR Eye from baseline to 12 months: In this case, ATRI remains high (**F**,**G**), and total vascular density experiences a decrease at the inferonasal subfield (**I**,**J**). SCP segmentation was not manually modified in any case. (**C**,**H**) show scanned area at both timepoints.

Sixteen eyes (13 patients) classified as active at baseline achieved quiescence at 12 months (A-I, 26.7%). Notably, these eyes received a higher number of intravitreal dexamethasone implants (DEX) both before entering the study (A-I: *n* = 9; 56.2% vs. A-A: *n* = 5; 29.4% vs. I-I: *n* = 6; 22.2%) and during the study period (A-I: *n* = 4, 25% vs. A-A: *n* = 2, 11.7% vs. I-I: *n* = 0) (see Supplemental Table S1, which shows previous and ongoing treatments). As anticipated, A-I eyes exhibited a greater decrease in FA score, MV, and ATRI at 12 months compared with the entire population (Table 1 and Table 2). Qualitative microvascular characterization in A-I eyes showed a higher proportion with perifoveal capillary looping at baseline and a significant reversal of looping and capillary irregularity at 12 months (Figure 1), in contrast to the entire population. Notably, A-I eyes demonstrated an increase in vascular density and perfusion at 12 months across all retinal fields studied, particularly pronounced in the inferonasal field (Figure 2 and Table 3). This differed from groups A-A and I-I, which exhibited a decline in vascular density and perfusion at 12 months. However, these distinctive changes were not statistically significant (see Supplemental Tables S2 and S3, which show vascular density and perfusion at fundus and superonasal field).

### 3.2. Active-Active Subgroup

Seventeen eyes (12 patients) classified as active at baseline remained active at 12 months (A-A, 28.3%) and exhibited distinctive characteristics, notably a higher cumulated dose of oral prednisone at baseline and more individuals still taking oral prednisone at 12 months. Intriguingly, A-A eyes were characterized by consistently showing 4–20 spots and never displaying ≤ 3 spots on the UWF^®^ exam at both timepoints. Persistent macular edema, as evidenced by higher CMT, MV, the presence of intraretinal fluid, and a thicker retina at the fundus (ATRI) and nasal field (NRT), characterized A-A eyes throughout the study. Additionally, there was an increase in retinal NV and a higher incidence of a damaged ellipsoid layer at the nasal subfield at 12 months in the A-A group. A-A eyes demonstrated higher perfusion and vascular density at baseline, although this characteristic was statistically significant only in the inferonasal subfield, not at the fundus or superonasal subfields.

### 3.3. Inactive-Inactive Subgroup

Twenty-seven eyes (18 patients) were consistently classified as inactive both at baseline and final follow-up (I-I, 45%). This group exhibited a significantly longer disease evolution time than the entire population. Notably, there were fewer eyes with 4–20 spots and a trend toward >20 medium-sized spots at baseline (see Supplemental Table S4, which shows UWF^®^ spots’ features). As expected, most inflammatory parameters were lower in the I-I group at both timepoints, including FA score, CMT, and ATRI. Minimal changes in these parameters were observed during the follow-up. This trend was also evident in the retinal nasal fields (NRT and NV). Conversely, the I-I group exhibited higher atrophy markers (AARI) and more eyes with a damaged or absent ellipsoid layer. Concerning microvascular features, lower vascular density and perfusion typically characterized these eyes, with significance noted in the inferonasal subfield but not in other retinal subfields. As mentioned earlier, a generalized decrease in vascular density and perfusion was observed in the I-I group from baseline.

Interestingly, changes in choroidal thickness were not characteristic of any particular group nor predictive of any outcome, with quite wide interindividual variability in all BSCR groups.

**Figure 3:**
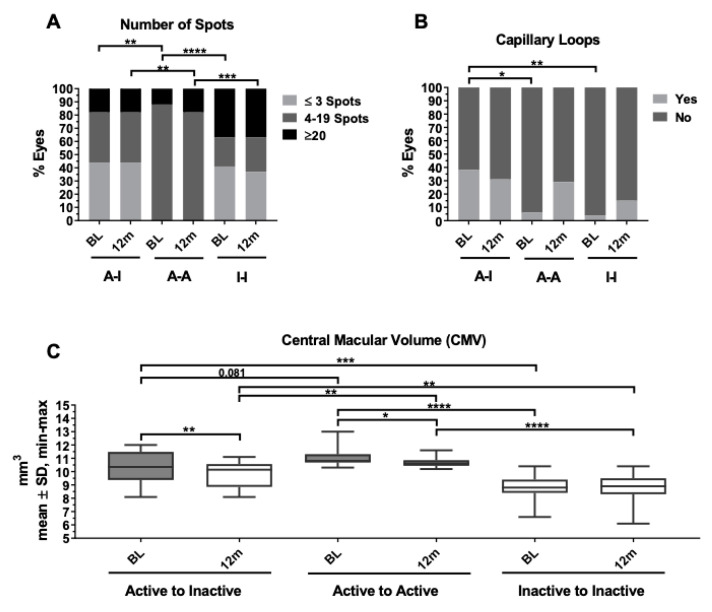
Comparison of the number of spots in UWF pseudocolor images (**A**), presence of perifoveal capillary loops (**B**), and central macular volume (**C**) among birdshot chorioretinitis outcome groups. (**A**) Note that active eyes accounting for 4–19 spots at baseline remained often active at 12 months. Conversely, active eyes showing ≤3 spots reached quiescence. (**B**) Note that active eyes with capillary loops at baseline reached quiescence at 12 months. Conversely, those active eyes without looping remained active. (**C**) Higher macular volume at baseline was found in those active eyes which remained active at 12 months. * p < 0.05, ** p < 0.01, *** p < 0.001, **** p < 0.0001.

**Figure 4:**
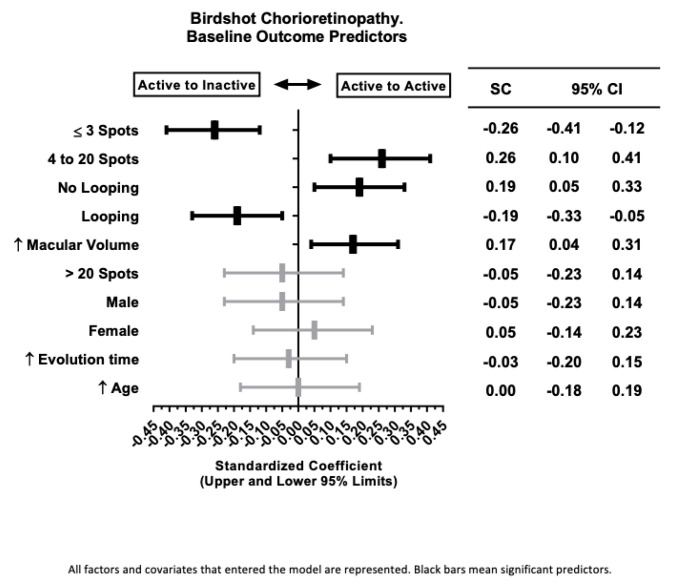
Partial least square discriminant analysis (PLS-DA) of factors and covariates predicting activity outcomes in birdshot chorioretinitis. ↑ = more.

When examining potential outcome predictors in active eyes, the univariate analysis between baseline variables in the A-I vs. A-A groups revealed significant differences in the number of spots (*p* = 0.004), MV (*p* = 0.081), and the presence of perifoveal capillary loops (*p* = 0.039) (Figure 3). Local and systemic corticosteroid treatments did not significantly influence the outcome of active eyes. Clinical variables such as age, sex, and evolution time to baseline were also included in the model due to their clinical relevance. Following PLS-DA modeling, active eyes with BSCR presenting ≤3 spots or perifoveal capillary looping in the SCP were more prone to reach quiescence at 12 months. Conversely, eyes with 4 to 20 spots, lacking capillary looping, or with a higher MV at baseline were identified as having a higher risk of remaining active at the 12-month follow-up (Figure 4).

## 4. Discussion

BSCR manifests as chronic and progressive bilateral posterior uveitis, affecting both stromal choroid and retinal tissues. Retinal involvement is marked by extensive capillaritis and diffuse phlebitis, eventually progressing to extensive retinal atrophy over time [6,7]. Therapeutic management of BSCR poses challenges due to its unpredictable course. Clinically, BSCR presents in various forms, including primary progressive forms, chronic recurrent forms, and a self-limited type [8]. The unpredictable nature of the disease underscores the need for diverse therapeutic approaches. Understanding the pathophysiological mechanisms of BSCR is crucial for exploring novel therapeutic strategies in this otherwise unpredictable condition.

OCT-A provided intriguing insights into BSCR pathophysiology; microvascular characterization using OCT-A suggests chronic retinal ischemia in active BSCR, evident through diminished vascular density and perfusion at the 12-month follow-up. Interestingly, in eyes that transitioned to quiescence (A-I), both vascular density and perfusion tended to increase, suggesting that when inflammation is reduced and under control, capillary reperfusion occurs throughout the retina. The observed reversal of capillary “closure” during the active inflammation stage to near-normal values may have implications in preventing sustained retinal ischemia and subsequent irreversible retinal atrophy. Cross-sectional studies by other authors indicated a decrease in capillary density at the superficial capillary plexus in inactive BSCR compared with healthy subjects. Speculation arises that ischemia in this plexus might contribute to the damage of ganglion cells and their axons, potentially leading to macular thinning in BSCR [17]. As per previous findings [18], our study supports these observations, noting a significant decrease in vascular density in the full retina in BSCR patients compared with healthy controls.

Inflammation in the BSCR affects the retina and choroid in both a bifocal, and probably, independent manner. Notably, our study extends beyond the macular area, revealing that these microvascular changes are more pronounced in the inferonasal subfield, coinciding with more intense choroidal involvement in this region.

Roberts et al. found that BCVA eliminate negatively correlated with the vascular density of the SCP, DCP, and the whole retina in patients with BSCR [18]. In our study, BCVA did not characterize any particular group nor was predictive of the inflammatory outcome (Table S1).

Our investigation reveals that specific abnormalities in the perifoveal superficial capillary network, particularly capillary looping, could serve as predictive indicators of short-term quiescence in BSCR. Previous studies by different authors utilizing OCT-A observed capillary loops in the macular microvasculature of BSCR patients, with varying frequencies of 88%, 58%, 58.3%, and 63.6% [12,17,19,20]. Additionally, Pohlmann et al. noted capillary irregularities in 53% of patients [19]. In our study, these two microvascular abnormalities exhibited a significant decrease in eyes that reached quiescence (A-I) at the 12-month timepoint. An opposite non-significant trend was observed in the A-A and I-I groups as compared to A-I, by increasing the proportion of eyes with capillary loops at 12 months. This could mean a potential underlying activity in some eyes, which might benefit from treatment, even if clinically judged as inactive.

De Carlo et al. speculate that these retinal microvascular changes may cause relative ischemia that could be pro-angiogenic, even in the absence of areas of no capillary perfusion [12]. This would agree with our study, where we have observed that when we reach quiescence, there is capillary reperfusion, and these microvascular abnormalities may reverse. These findings underscore the potential utility of monitoring capillary looping and irregularity in the perifoveal superficial capillary network through OCT-A as predictive markers of quiescence in BSCR.

ICG is essential to detect choroidal inflammation and to precisely know the number of choroidal spots (dark dots) [2]; however, in this study, we studied the number of spots observed in the UWF pseudocolor images, which may reflect more chronic choroidal foci than those revealed only via ICG. Conventional BSCR assessment has relied on typical funduscopic lesions as a benchmark for disease evaluation. The natural history of these lesions has been delineated, starting as a few, poorly defined, non-pigmented lesions primarily located in the inferior peripapillary region. As the disease advances, these lesions tend to become more widespread, larger, well-defined, pigmented, and ultimately atrophic [21,22]. We noted that achieving quiescence at the 12-month mark was more frequent in BSCR eyes with mild retinochoroidal involvement, characterized by three or fewer BSCR spots at baseline. Conversely, eyes with moderate retinochoroidal involvement, displaying between 4 and 20 spots, were more likely to remain active. These findings suggest that mild choroidal involvement is associated with better clinical outcomes, or that early intervention is perhaps the most effective strategy to interrupt the progression of most clinical BSCR forms.

Despite our investigation, no significant differences were identified in evolution time or systemic treatment modalities between A-I and A-A at the 12-month mark, suggesting that factors beyond those examined in our study might play a crucial role in determining outcomes. One potential influential factor not explored herein is the presence of *ERAP* genetic variants, in homozygosity or heterozygosity. Recent studies proposed that such genetic variations could impact the natural history of BSCR. The intricate interplay of these unknown variables underscores the need for further research to unravel the comprehensive landscape influencing BSCR outcomes [23].

The I-I subgroup was characterized by a longer evolution time, with lower inflammatory parameters and higher atrophy (AARI) at baseline, suggesting a possible burnt-out status. Whether additional immunomodulation could stop or reverse the observed vascular density decline in this presumed clinically inactive group needs further research.

Inflammatory activity in BSCR does tend to align with inflammatory signs in the retinal vasculature, as evidenced by FA [19]. Consistent with our study, eyes that achieved quiescence at 12 months (A-I) demonstrated a significant decrease in the average FA score. Another noteworthy observation involves the thickening of the temporal vascular arches (averaged thickened retinal index, ATRI), as depicted in the thickness map, serving as a valuable indicator of inflammatory activity. Some authors correlated this perivascular thickening with vasculitis in FA [24]; others deemed this perivascular thickening in OCT a non-invasive biomarker of inflammation, correlating with MV, CMT, and even vitreous haze [25]. In our study, we employed high-resolution color all-retina thickness maps, processing and quantifying the data, revealing a significant decrease in the ATRI and an increase in the AARI in eyes that reached quiescence (A-I) at the 12-month mark. These findings underscore the potential of these indices as valuable markers for monitoring inflammatory activity and assessing treatment outcomes in BSCR. Despite an increase in VD and PI observed in active eyes after inactivation at 12 months, certain grade of atrophy was found in parallel (increase in AARI), which means that active inflammation may lead to permanent tissue damage and retinal atrophy if not promptly and adequately managed.

Structural OCT stands out as an excellent method for analyzing retinal layers, widely employed in studying macular edema and proving highly useful for assessing macular atrophy and photoreceptor disruption [2,22,26]. In our study, a higher MV at baseline emerged as a predictor, indicating a higher risk of remaining active at the 12-month follow-up. Active eyes (A-A) were further characterized by higher CMT and the presence of intraretinal fluid, aligning with previous reports of a high prevalence of macular edema in BSCR, reaching up to 63% [27]. Interestingly, at the nasal field, a typical location for BSCR spots, NRT and NV were also elevated in A-A, showing a significant decrease in those that transitioned to quiescence at 12 months (A-I). These findings highlight the utility of structural OCT in capturing critical parameters indicative of BSCR progression and treatment response in the fundus and nasal regions.

The disruption of the ellipsoid layer constitutes a significant contributor to visual loss in BSCR, reported in up to 33% of cases, with instances of reversibility noted after systemic treatment [26]. In our study, while no significant differences were identified at the macula level among subgroups, the inferonasal level displayed an increasing trend for ellipsoid disruption in A-A eyes and a decrease in A-I eyes. Parallel findings from Keane et al. demonstrated a generalized disruption of the ellipsoid layer in 54% of BSCR eyes at the nasal extramacular level. This underscores the importance of OCT imaging beyond the macula, suggesting that structural changes may manifest outside the macula even when no observable alterations are evident at the macular level [28].

## 5. Conclusions

In conclusion, our study revealed a widespread augmentation in vascular density and perfusion at the superficial capillary retinal plexus, particularly pronounced in the inferonasal field, following the inactivation of BSCR. Additionally, the presence of perifoveal capillary looping demonstrated potential as a predictive factor for achieving effective short-term inflammatory control. We must acknowledge that further investigation and external validation with a larger patient cohort are imperative to draw more definitive and compelling conclusions.

## Figures and Tables

**Table 1:** Clinical inflammation scores, tomographic, and angiographic characterizing variables in birdshot chorioretinitis with different activity outcomes.

	Active-Inactive		*p*-Value	Active-Active		*p*-Value	Inactive-Inactive		*p*-Value
	*n*	%		*n*	%		*n*	%	
Patients	13	43.3		12	40.0		18	60.0	
Eyes	16	26.7		17	28.3		27	45.0	
	mean	±SD		mean	±SD		mean	±SD	
Age (years)	58.70	11.10	0.692	58.90	14.80	0.754	60.90	10.90	0.525
Evolution Time (months)	97.50	49.90	0.331	92.00	51.40	0.158	132.20	77.80	**0.032**
Inflammation VH (NEI)	*n*	%		*n*	%		*n*	%	
BL 0+	13	81.25	0.345	14	82.35	0.401	26	96.30	0.100
12 m 0+	15	93.75	0.533	17	100.00	0.510	26	96.30	0.900
∆ 12 m − BL	2.00	12.50	0.533	3.00	17.65	0.510	0.00	0.00	0.157
BL 0.5+/1+	3	18.75	0.345	3	17.65	0.401	1	3.70	0.100
12 m 0.5+/1+	1	6.25	0.533	0	0.00	0.510	1	3.70	0.900
∆ 12 m − BL	−2.00	−12.50	0.345	−3.00	−17.65	0.401	0.00	0.00	0.157
UWF Imaging: *n* Spots	*n*	%		*n*	%		*n*	%	
BL ≤ 3	7	43.75	0.185	0	0.00	**0.001**	11	40.74	0.114
12 m ≤ 3	7	43.75	0.133	0	0.00	**0.001**	10	37.04	0.194
∆ 12 m − BL	0	0	0.534	0	0	0.567	−1	−4	0.900
BL 4–20	6	37.50	0.502	15	88.24	**<0.0001**	6	22.22	**0.002**
12 m 4–20	6	37.50	0.502	14	82.35	**<0.0001**	7	25.93	**0.009**
∆ 12 m − BL	0	0	0.534	−1	−6	0.567	1	4	0.900
BL > 20	3	18.75	0.534	2	11.77	0.149	10	37.04	0.052
12 m > 20	3	18.75	0.433	3	17.65	0.347	10	37.04	0.115
∆ 12 m − BL	0	0	0.534	1	6	0.567	0	0	0.900
UWF FA Score	*n*	%		*n*	%		*n*	%	
BL > 5	11	68.75	0.164	11	64.71	0.286	10	37.04	**0.026**
12 m > 5	10	62.50	0.332	12	70.59	0.074	9	33.33	**0.012**
∆ 12 m − BL	−1	−6	0.991	1	6	0.830	−1	−4	0.509
	mean	±SD		mean	±SD		mean	±SD	
BL FA Score	10.06	5.02	0.068	9.94	5.92	0.072	5.26	4.43	**0.001**
12 m FA Score	6.25	4.78	0.778	7.53	4.43	0.105	4.82	4.39	0.085
∆ 12 m − BL	−4	5.30	**0.026**	−2	3.37	0.534	0	2.60	**0.011**
OCT Macular Cube	mean	±SD		mean	±SD		mean	±SD	
BL CMT (μm)	271.38	59.96	0.158	285.12	82.96	**0.014**	217.30	37.44	**0.000**
12 m CMT (μm)	243.63	37.54	0.595	268.71	54.77	**0.002**	215.41	37.95	**0.001**
∆ 12 m − BL	−27.75	42.51	0.106	−16.41	65.01	0.691	−1.89	6.52	0.073
BL MV (mm^3^)	10.39	1.17	0.088	11.10	0.66	**<0.0001**	8.86	0.82	**<0.0001**
12 m MV (mm^3^)	9.78	0.92	0.462	10.71	0.40	**<0.0001**	8.80	0.90	**<0.0001**
∆ 12 m − BL	−0.61	0.62	**0.020**	−0.39	0.70	0.479	−0.06	0.42	**0.007**
	*n*	%		*n*	%		*n*	%	
BL IRF	4	25.00	0.571	6	35.29	0.085	2	7.41	**0.031**
12 m IRF	1	6.25	0.795	4	23.53	**0.021**	0	0.00	**0.043**
∆ 12 m − BL	−3	−19	0.345	−2	−12	0.957	−2	−7	0.393

The *p*-value indicates the deviation from the entire population in the characterization of the A-I, A-A, and I-I groups with the respective characterizing variables. Lighter shades of grey signify a decrease, while darker shades denote an increase from baseline to 12 months of follow-up. *p* < 0.05 is marked in bold. Abbreviations: BL: baseline; m: months; VH: vitreous haze; FA: fluorescein angiography; CMT: central macular thickness; MV: macular volume; IRF: intraretinal fluid; UWF: ultra-wide field imaging.

**Table 2:** Tomographic characterizing variables in the fundus and inferonasal fields of birdshot chorioretinitis with different activity outcomes.

	Active-Inactive		*p*-Value	Active-Active		*p*-Value	Inactive-Inactive		*p*-Value
	*n*	%		*n*	%		*n*	%	
Eyes	16	26.7		17	28.3		27	45.0	
Macular Cube OCT	mean	±SD		mean	±SD		mean	±SD	
BL ATRI	65.93	45.89	0.070	82.36	37.99	**<0.0001**	17.96	13.53	**<0.0001**
12 m ATRI	30.27	24.25	0.556	64.75	25.81	**<0.0001**	16.66	12.26	**<0.0001**
∆ 12 m − BL	−35.66	39.83	**0.005**	−17.61	42.91	0.721	−1.30	7.37	**0.005**
BL AARI	75.19	47.50	**0.022**	57.02	26.10	**<0.0001**	142.70	26.27	**<0.0001**
12 m AARI	102.09	42.41	0.323	69.43	25.54	**<0.0001**	143.88	27.65	**<0.0001**
∆ 12 m − BL	26.90	25.54	**0.001**	12.41	24.94	0.798	1.18	10.40	**0.002**
Macular Ellipsoid:	*n*	%		*n*	%		*n*	%	
BL Normal	6	37.50	0.570	8	47.06	0.127	5	18.51	0.054
12 m Normal	8	50.00	0.118	8	47.06	0.177	4	14.81	**0.007**
∆ 12 m − BL	2	13	0.387	0	0	0.957	−1	−4	0.470
BL Damaged	9	56.25	0.727	9	52.94	0.499	8	66.67	0.357
12 m Damaged	7	43.75	0.186	9	52.94	0.607	19	70.37	0.097
∆ 12 m − BL	−2	−13	0.341	0	0	0.957	11	4	0.509
BL Absent	1	6.25	0.795	0	0.00	0.176	4	14.82	0.136
12 m Absent	1	6.25	0.795	0	0.00	0.176	4	14.82	0.136
∆ 12 m-BL	0	0	0.879	0	0	0.957	0	0	0.918
Nasal Field Cube OCT	mean	±SD		mean	±SD		mean	±SD	
BL NRT (μm)	231.56	31.67	0.191	240.29	29.75	**0.008**	205.74	25.47	**<0.001**
12 m NRT (μm)	204.04	54.08	0.251	231.12	22.45	**0.021**	207.48	27.30	0.281
∆ 12 m − BL	−27.52	66.84	**0.044**	−9.17	30.04	0.998	1.74	20.48	**0.073**
BL Nasal Volume (mm^3^)	8.11	1.35	**0.008**	7.28	1.51	0.798	6.94	0.94	**0.032**
12 m Nasal Volume (mm^3^)	7.63	0.78	0.145	7.59	1.26	0.183	6.89	1.03	**0.012**
∆12 m − BL	−0.48	0.85	**0.050**	0.31	1.32	0.066	−0.05	0.66	0.936
Nasal Ellipsoid:	*n*	%		*n*	%		*n*	%	
BL Normal	5	31.25	0.635	7	41.18	0.133	4	14.82	0.068
12 m Normal	7	43.75	0.060	4	23.53	0.892	4	14.82	0.111
∆12 m − BL	2	13	0.278	−3	−18	0.510	0	0	0.172
BL Damaged	10	62.50	0.930	8	47.06	0.117	20	74.07	0.130
12 m Damaged	8	50.00	0.218	11	64.71	0.903	19	70.37	0.324
∆12 m − BL	−2	−13	0.068	3	18	0.070	−1	−4	0.298
BL Absent	1	6.25	0.629	2	11.77	0.767	3	11.11	0.807
12 m Absent	1	6.25	0.491	2	11.77	0.957	4	14.82	0.524
∆12 m − BL	0	0	0.698	0	0	0.265	1	4	0.470

The *p*-value indicates the deviation from the entire population in the characterization of the A-I, A-A, and I-I groups with the respective characterizing variables. Lighter shades of grey signify a decrease, while darker shades denote an increase from baseline to 12 months of follow-up. *p* < 0.05 is marked in bold. Abbreviations: BL: baseline; m: months; ATRI: averaged thickness retinal index; AARI: averaged atrophy retinal index; NRT: nasal retinal thickness.

**Table 3:** Qualitative characterizing variables of OCT-A 3 × 3 frame in the macular area and quantitative microvascular indices in OCT-A 6 × 6 frame in the inferonasal area (superficial capillary plexus) of birdshot chorioretinitis with different activity outcomes.

	Active-Inactive		*p*-Value	Active-Active		*p*-Value	Inactive-Inactive		*p*-Value
	*n*	%		*n*	%		*n*	%	
Eyes	16	26.70		17	28.30		27	45.00	
Fundus field OCTA Qualitative Assessment	*n*	%		*n*	%		*n*	%	
BL Capillary Loops	6	37.50	**0.003**	1	5.88	0.327	1	3.70	0.056
12 m Capillary Loops	5	31.25	0.0405	5	29.41	0.500	4	14.82	0.175
∆ 12 m − BL	−1.00	−6.25	**0.048**	1.00	23.53	0.398	3.00	11.12	0.749
BL Telangiectasia	9	56.25	0.091	14	82.35	0.347	21	77.78	0.502
12 m Telangiectasia	12	75.00	0.884	10	58.82	0.133	22	81.48	0.214
∆ 12 m − BL	3.00	18.75	0.148	−4.00	−23.53	**0.039**	1.00	3.70	0.676
BL Increased IC spaces	14	87.50	0.879	15	88.24	0.957	24	88.89	0.918
12 m Increased IC spaces	14	87.50	0.879	14	82.35	0.401	25	92.59	0.393
∆ 12 m − BL	0.00	0.00	0.949	−1.00	−5.89	0.382	1.00	3.70	0.848
BL Capillary Irregularity	13	81.25	0.534	12	70.58	0.627	20	74.07	0.883
12 m Capillary Irregularity	9	56.25	0.060	13	76.47	0.892	23	85.19	0.111
∆ 12 m − BL	−4.00	−25.00	**0.016**	1.00	5.89	0.737	3.00	11.12	0.136
Inferonasal Field OCTA Vascular Density (VD) mm^−2^	mean	±SD		mean	±SD		mean	±SD	
BL Total VD	6.81	3.20	0.851	8.33	4.97	**0.043**	5.49	3.37	**0.046**
12 m Total VD	7.63	4.13	0.106	6.65	4.28	0.563	4.93	3.98	**0.050**
∆ 12 m − BL	0.82	4.36	0.134	−1.68	3.79	0.165	−0.55	3.86	0.939
BL Central VD	5.76	4.43	0.911	7.21	5.48	0.096	4.60	3.54	0.108
12 m Central VD	6.56	5.37	0.161	5.32	4.06	0.829	4.11	4.64	0.149
∆ 12 m − BL	0.81	6.46	0.255	−1.89	5.15	0.234	−0.49	4.79	0.946
BL Internal VD	6.10	3.73	0.954	8.05	5.14	**0.032**	4.99	3.52	0.059
12 m Internal VD	7.05	4.85	0.136	6.11	4.19	0.586	4.41	4.17	0.069
∆ 12 m − BL	0.95	5.37	0.115	−1.95	3.83	0.130	−0.57	3.83	0.978
BL External VD	7.09	3.09	0.770	8.46	4.95	0.051	5.67	3.42	**0.042**
12 m External VD	7.84	4.00	0.106	6.88	4.41	0.555	5.15	3.95	**0.049**
∆ 12 m − BL	0.75	4.19	0.162	−1.59	3.80	0.192	−0.52	3.98	0.952
Inferonasal Field OCTA Perfusion Index (PI)%	mean	±SD		mean	±SD		mean	±SD	
BL Total PI	16.19	7.97	0.844	20.36	12.48	0.203	14.85	16.32	0.328
12 m Total PI	18.33	10.11	0.106	16.12	10.82	0.499	11.51	9.79	**0.040**
∆ 12 m − BL	2.14	10.73	0.135	−4.25	9.46	0.441	−3.34	15.83	0.528
BL Central PI	13.64	10.93	0.978	17.70	13.33	0.074	10.94	8.72	0.101
12 m Central PI	15.46	13.12	0.175	12.29	9.64	0.913	9.81	11.59	0.192
∆ 12 m − BL	1.82	16.38	0.251	−5.41	12.49	0.172	−1.13	11.80	0.828
BL Internal PI	14.46	9.30	0.894	19.91	13.00	**0.021**	11.72	8.53	**0.048**
12 m Internal PI	17.00	12.00	0.150	15.16	10.73	0.477	10.38	10.25	0.054
∆ 12 m − BL	2.54	13.44	0.109	−4.75	9.69	0.126	−1.34	9.29	0.967
BL External PI	16.84	7.70	0.785	20.64	12.41	**0.034**	13.14	8.27	**0.031**
12 m External PI	18.81	9.81	0.103	16.56	11.15	0.504	11.97	9.68	**0.040**
∆ 12 m − BL	1.97	10.29	0.150	−4.08	9.57	0.161	−1.17	9.64	0.992

The *p*-value indicates the deviation from the entire population in the characterization of the A-I, A-A, and I-I groups with the respective characterizing variables. Lighter shades of grey signify a decrease, while darker shades denote an increase from baseline to 12 months of follow-up. *p* < 0.05 is marked in bold. Abbreviations: BL: baseline, m: months; IC: intercapillary.

## Data Availability

The original contributions presented in the study are included in the article/Supplementary Materials, further inquiries can be directed to the corresponding authors.

## Supplementary Materials

The following supporting information can be downloaded at: https://www.mdpi.com/article/10.3390/biomedicines12102414/s1, Table S1: Demographics, previous and ongoing treatments, and visual acuity in Birdshot chorioretinitis with different activity outcomes. Table S2: Quantitative characterizing microvascular indices in 6 × 6 frame OCT-A (superficial capillary plexus) at the fundus field of Birdshot chorioretinitis with different activity outcomes. Table S3: Quantitative characterizing microvascular indices in 6 × 6 frame OCT-A (superficial capillary plexus) at the superonasal field of Birdshot chorioretinitis with different activity outcomes. Table S4: Ultra-WideField^®^ spots’ features characterize Birdshot chorioretinitis eyes with different activity outcomes.

## References

[1] Pagnoux C., Mahr A., Aouba A., Bérezné A., Monnet D., Cohen P., Levinson R.D., Brézin A.P., Guillevin L. (2010). Extraocular Manifestations of Birdshot Chorioretinopathy in 118 French Patients. Presse Med..

[2] Bousquet E., Duraffour P., Debillon L., Somisetty S., Monnet D., Brézin A.P. (2022). Birdshot Chorioretinopathy: A Review. J. Clin. Med..

[3] Levinson R.D., Rajalingam R., Park M.S., Reed E.F., Gjertson D.W., Kappel P.J., See R.F., Rao N.A., Holland G.N. (2004). Human Leukocyte Antigen A29 Subtypes Associated with Birdshot Retinochoroidopathy. Am. J. Ophthalmol..

[4] Brézin A.P., Monnet D., Cohen J.H.M., Levinson R.D. (2011). HLA-A29 and Birdshot Chorioretinopathy. Ocul. Immunol. Inflamm..

[5] Herbort C.P., Pavésio C., LeHoang P., Bodaghi B., Fardeau C., Kestelyn P., Neri P., Papadia M. (2017). Why Birdshot Retinochoroiditis Should Rather Be Called “HLA-A29 Uveitis”?. Br. J. Ophthalmol..

[6] Monnet D., Brézin A.P. (2006). Birdshot Chorioretinopathy. Curr. Opin. Ophthalmol..

[7] The Standardization of Uveitis Nomenclature (SUN) Working Group (2021). Classification Criteria for Birdshot Chorioretinitis. Am. J. Ophthalmol..

[8] Lages V., Skvortsova N., Jeannin B., Gasc A., Herbort C.P. (2019). Low-Grade “Benign” Birdshot Retinochoroiditis: Prevalence and Characteristics. Int. Ophthalmol..

[9] Gordon L.K., Goldhardt R., Holland G.N., Yu F., Levinson R.D. (2006). Standardized Visual Field Assessment for Patients with Birdshot Chorioretinopathy. Ocul. Immunol. Inflamm..

[10] Thorne J.E., Jabs D.A., Kedhar S.R., Peters G.B., Dunn J.P. (2008). Loss of Visual Field Among Patients with Birdshot Chorioretinopathy. Am. J. Ophthalmol..

[11] Holder G.E., Robson A.G., Pavesio C., Graham E.M. (2005). Electrophysiological Characterisation and Monitoring in the Management of Birdshot Chorioretinopathy. Br. J. Ophthalmol..

[12] De Carlo T.E., Bonini Filho M.A., Adhi M., Duker J.S. (2015). Retinal and Choroidal Vasculature in Birdshot Chorioretinopathy Analyzed Using Spectral Domain Optical Coherence Tomography Angiography. Retina.

[13] Böni C., Thorne J.E., Spaide R.F., Ostheimer T.A., Sarraf D., Levinson R.D., Goldstein D.A., Rifkin L.M., Vitale A.T., Jaffe G.J. (2017). Multimodal Imaging of the Disease Progression of Birdshot Chorioretinopathy. Ocul. Immunol. Inflamm..

[14] Levinson R.D., Brezin A., Rothova A., Accorinti M., Holland G.N. (2006). Research Criteria for the Diagnosis of Birdshot Chorioretinopathy: Results of an International Consensus Conference. Am. J. Ophthalmol..

[15] Tugal-Tutkun I., Herbort C.P., Khairallah M., Allegri P., Biziorek B., Bodaghi B., Bouchenaki N., Cimino L., Fardeau C., Gupta A. (2010). Scoring of Dual Fluorescein and ICG Inflammatory Angiographic Signs for the Grading of Posterior Segment Inflammation (Dual Fluorescein and ICG Angiographic Scoring System for Uveitis). Int. Ophthalmol..

[16] Llorenç V., Serrano A.R., Mesquida M., Lin P., Esquinas C., Sainz-de-la-Maza M., Metea C., Bosch A., Calvo M., Balter A. (2021). Swept-Source Optical Coherence Tomography Objective Composite Activity Score for Uveitis. Acta Ophthalmol..

[17] Pichi F., Lembo A., Nucci P., Neri P. (2023). Optical Coherence Tomography Angiography in Birdshot Chorioretinopathy. Eur. J. Ophthalmol..

[18] Roberts P.K., Nesper P.L., Goldstein D.A., Fawzi A.A. (2018). Retinal Capillary Density in Patients with Birdshot Chorioretinopathy. Retina.

[19] Pohlmann D., Macedo S., Stübiger N., Pleyer U., Joussen A.M., Winterhalter S. (2017). Multimodal Imaging in Birdshot Retinochoroiditis. Ocul. Immunol. Inflamm..

[20] Forte R., Saleh M., Aptel F., Chiquet C. (2020). Evaluation of Photoreceptors, Retinal Capillary Plexuses, and Choriocapillaris in Patients With Birdshot Chorioretinopathy. Retina.

[21] Monnet D., Brézin A.P., Holland G.N., Yu F., Mahr A., Gordon L.K., Levinson R.D. (2006). Longitudinal Cohort Study of Patients with Birdshot Chorioretinopathy. I. Baseline Clinical Characteristics. Am. J. Ophthalmol..

[22] Minos E., Barry R.J., Southworth S., Folkard A., Murray P.I., Duker J.S., Keane P.A., Denniston A.K. (2016). Birdshot Chorioretinopathy: Current Knowledge and New Concepts in Pathophysiology, Diagnosis, Monitoring and Treatment. Orphanet J. Rare Dis..

[23] Kuiper J.J.W., Van Setten J., Ripke S., Van ’T Slot R., Mulder F., Missotten T., Baarsma G.S., Francioli L.C., Pulit S.L., De Kovel C.G.F. (2014). A Genome-Wide Association Study Identifies a Functional ERAP2 Haplotype Associated with Birdshot Chorioretinopathy. Hum. Mol. Genet..

[24] Knickelbein J.E., Tucker W., Kodati S., Akanda M., Sen H.N. (2018). Non-Invasive Method of Monitoring Retinal Vasculitis in Patients with Birdshot Chorioretinopathy Using Optical Coherence Tomography. Br. J. Ophthalmol..

[25] Thomas A.S., Hatef A.L., Stinnett S.S., Keenan R.T., Jaffe G.J. (2019). Perivascular Thickening on Optical Coherence Tomography as a Marker of Inflammation in Birdshot Retinochoroiditis. Retina.

[26] Teussink M.M., Huis in het Veld P.I., de Vries L.A.M., Hoyng C.B., Klevering B.J., Theelen T. (2016). Multimodal Imaging of the Disease Progression of Birdshot Chorioretinopathy. Acta Ophthalmol..

[27] Rothova A., Berendschot T.T.J.M., Probst K., Van Kooij B., Baarsma G.S. (2004). Birdshot Chorioretinopathy: Long-Term Manifestations and Visual Prognosis. Ophthalmology.

[28] Keane P.A., Allie M., Turner S.J., Southworth H.S., Sadda S.R., Murray P.I., Denniston A.K. (2013). Characterization of Birdshot Chorioretinopathy Using Extramacular Enhanced Depth Optical Coherence Tomography. JAMA Ophthalmol..

