# Supplementary material for: Microvascular and Structural Characterization of Birdshot Chorioretinitis in Active and Inactive Phases
Source: Biomedicines. 2024 Oct 21;12(10):2414. doi: 10.3390/biomedicines12102414 (PMC11505013; doi:10.3390/biomedicines12102414)
Supplement: Supplementary file 1 [file biomedicines-12-02414-s001.zip › Table S1.pdf]

Table S1. Demographics, previous and ongoing treatments, and visual acuity in Birdshot chorioretinitis with different activity outcomes.

	ACTIVE-INACTIVE		*p*-value	ACTIVE-ACTIVE		*p*-value	INACTIVE-INACTIVE		*p*-value
	**n**	**%**		**n**	**%**		**n**	**%**	
**Patients**	13	43.3		12	40.0		18	60.0	
**Eyes**	16	26.7		17	28.3		27	45.0	
**Laterality: Right Eye**	6	37.50	0.262	7	41.18	0.410	17	62.96	0.078
**Previous Treatments**	**n**	**%**		**n**	**%**		**n**	**%**	
PPV	1	6.25	0.991	1	5.88	0.936	2	7.41	0.848
DEX	9	56.25	**0.032**	5	29.41	0.708	6	22.22	0.109
Cumulated PDN (mg)	1188.75	1269.51	1.000	2011.77	2940.32	**0.032**	670.00	711.38	0.051
	**mean**	**±SD**		**mean**	**±SD**		**mean**	**±SD**	
Time IMT/Evol (m)	0.26	0.27	0.469	0.35	0.38	0.706	0.33	0.42	0.763
Time BIO/Evol (m)	0.27	0.29	0.955	0.29	0.32	0.675	0.25	0.32	0.667
Time IMT or BIO/Evol	0.53	0.39	0.535	0.64	0.39	0.505	0.58	0.43	0.958
**Ongoing Treatments**	**n**	**%**		**n**	**%**		**n**	**%**	
BL DEX	2	12.50	0.187	0	0.00	0.361	1	3.70	0.735
12 m DEX	4	25.00	**0.042**	2	11.77	0.767	0	0.00	**0.022**
Δ 12 m - BL	*2*	*13*	*0.304*	*2*	*12*	*0.334*	*-1*	*-4*	*0.073*
BL Systemic CS	6	37.50	0.205	7	41.18	0.089	2	7.41	**0.005**
12 m Systemic CS	5	31.25	0.761	9	52.94	**0.013**	3	11.11	**0.008**
Δ 12 m - BL	*-1*	*-6*	*0.187*	*2*	*12*	*0.152*	*1*	*4*	*0.289*
BL Classic IMT	3	18.75	0.225	2	11.77	0.767	1	3.70	0.172
12 m Classic IMT	3	18.75	0.482	2	11.77	0.866	3	11.11	0.676
Δ 12 m - BL	*0*	*0*	*0.698*	*0*	*0*	*0.767*	*2*	*7*	*0.298*
BL Biotherapy	3	18.75	0.433	7	41.18	0.133	6	22.22	0.502
12 m Biotherapy	5	31.25	0.623	9	52.94	0.117	8	29.63	0.324
Δ 12 m - BL	*2*	*13*	*0.698*	*2*	*12*	*0.767*	*2*	*7*	*0.698*
**Lens Status**	**n**	**%**		**n**	**%**		**n**	**%**	
BL Cataract	2	12.50	0.649	4	23.52	0.398	4	14.81	0.749
12 m Cataract	2	12.50	0.147	7	41.18	0.133	7	25.93	0.914
Δ 12 m - BL	*0*	*0*	*0.949*	*3*	*18*	*0.717*	*3*	*11*	*0.550*
BL Clear	10	62.50	0.826	11	64.71	0.659	15	55.56	0.539
12 m Clear	10	62.50	0.262	8	47.06	0.784	12	44.44	0.452
Δ 12 m - BL	*0*	*0*	*0.949*	*-3*	*-18*	*0.401*	*-3*	*-11*	*0.918*
**BCVA (LogMAR)**	**mean**	**±SD**		**mean**	**±SD**		**mean**	**±SD**	
BL BCVA	0.42	0.72	0.383	0.29	0.35	0.748	0.29	0.41	0.627
12 m BCVA	0.34	0.51	0.750	0.25	0.30	0.566	0.32	0.57	0.813
Δ 12 m - BL	*-0.08*	*0.28*	*0.199*	*-0.04*	*0.12*	*0.591*	*0.03*	*0.21*	*0.103*

The *p*-value indicates the deviation from the entire population in the characterization of the A-I, A-A, and I-I groups with the respective characterizing variables. Lighter shades of grey signify a decrease, while darker shades denote an increase from baseline to 12 months of follow-up. $p < 0.05$ is marked in bold. Abbreviations: BL: baseline; m: months; PPV: pars plana vitrectomy; DEX: intravitreal Dexamethasone implant; PDN: prednisone; IMT: classic synthetic immunomodulatory therapy; BIO: biotherapy; BCVA: best correct visual acuity.
