# Supplementary material for: Microvascular and Structural Characterization of Birdshot Chorioretinitis in Active and Inactive Phases
Source: Biomedicines. 2024 Oct 21;12(10):2414. doi: 10.3390/biomedicines12102414 (PMC11505013; doi:10.3390/biomedicines12102414)
Supplement: Supplementary file 1 [file biomedicines-12-02414-s001.zip › Table S2.pdf]

Table S2. Quantitative characterizing microvascular indices in 6 × 6 frame OCT-A (superficial capillary plexus) at the fundus field of Birdshot chorioretinitis with different activity outcomes.

**Fundus Field OCTA**	**ACTIVE-INACTIVE**		***p*-value**	**ACTIVE-ACTIVE**		***p*-value**	**INACTIVE-INACTIVE**		***p*-value**
	**n**	**%**		**n**	**%**		**n**	**%**	
**Eyes**	16	26.70		17	28.30		27	45.00	
**Vascular Density (VD) mm-²**	**mean**	**±SD**		**mean**	**±SD**		**mean**	**±SD**	
BL Total VD	13.38	2.96	0.496	14.45	2.95	0.394	13.79	3.65	0.868
12 m Total VD	14.09	2.99	0.206	13.20	4.21	0.857	12.36	3.73	0.198
Δ 12 m - BL	*0.71*	*3.64*	***0.041***	*-1.25*	*3.34*	*0.532*	*-1.43*	*3.06*	*0.211*
BL Central VD	6.21	2.96	0.841	6.58	3.78	0.486	5.64	3.79	0.419
12 m Central VD	6.06	3.38	0.945	7.00	4.61	0.239	5.34	3.99	0.259
Δ 12 m - BL	*-0.15*	*3.69*	*0.889*	*0.42*	*3.40*	*0.462*	*-0.30*	*2.51*	*0.588*
BL Internal VD	12.94	3.02	0.507	13.92	3.59	0.531	13.47	3.85	0.981
12 m Internal VD	13.27	3.49	0.433	12.81	4.78	0.776	11.98	4.11	0.339
Δ 12 m - BL	*0.32*	*4.66*	*0.170*	*-1.12*	*4.00*	*0.799*	*-1.49*	*3.70*	*0.323*
BL External VD	13.79	3.11	0.503	14.92	2.83	0.347	14.15	3.67	0.797
12 m External VD	14.61	2.88	0.157	13.56	4.13	0.907	12.74	3.73	0.173
Δ 12 m - BL	*0.82*	*3.42*	***0.024***	*-1.36*	*3.25*	*0.418*	*-1.41*	*2.99*	*0.205*
**Perfussion Index (PI) %**	**mean**	**±SD**		**mean**	**±SD**		**mean**	**±SD**	
BL Total PI	31.94	7.72	0.495	34.95	7.92	0.334	32.89	9.44	0.788
12 m Total PI	33.75	7.77	0.220	31.91	10.80	0.716	29.19	9.63	0.155
Δ 12 m - BL	*1.81*	*9.65*	***0.047***	*-3.05*	*8.60*	*0.590*	*-3.70*	*8.06*	*0.201*
BL Central PI	13.34	6.63	0.991	14.86	8.95	0.380	12.43	8.52	0.432
12 m Central PI	13.41	7.55	1.000	16.05	10.78	0.179	11.74	9.26	0.223
Δ 12 m - BL	*0.06*	*8.28*	*0.990*	*1.18*	*8.13*	*0.451*	*-0.69*	*5.88*	*0.488*
BL Internal PI	30.41	7.78	0.454	33.23	9.13	0.486	31.95	9.87	0.972
12 m Internal PI	31.36	8.71	0.467	30.69	12.02	0.651	28.09	10.38	0.291
Δ 12 m - BL	*0.94*	*11.81*	*0.166*	*-2.54*	*10.12*	*0.877*	*-3.86*	*9.48*	*0.275*
BL External PI	33.15	8.15	0.516	36.24	7.70	0.309	33.95	9.59	0.731
12 m External PI	35.26	7.69	0.142	32.90	10.67	0.711	29.83	9.84	0.101
Δ 12 m – BL	*2.11*	*9.29*	***0.023***	*-3.34*	*8.39*	*0.551*	*-4.12*	*8.03*	*0.141*

The *p*-value indicates the deviation from the entire population in the characterization of the A-I, A-A, and I-I groups with the respective characterizing variables. Lighter shades of grey signify a decrease, while darker shades denote an increase from baseline to 12 months of follow-up. $p < 0.05$ is marked in bold. Abbreviations: OCTA: Optical coherence tomography angiography; BL: baseline; m: months.
