# Supplementary material for: Microvascular and Structural Characterization of Birdshot Chorioretinitis in Active and Inactive Phases
Source: Biomedicines. 2024 Oct 21;12(10):2414. doi: 10.3390/biomedicines12102414 (PMC11505013; doi:10.3390/biomedicines12102414)
Supplement: Supplementary file 1 [file biomedicines-12-02414-s001.zip › Table S3.pdf]

Table S3. Quantitative characterizing microvascular indices in 6 × 6 frame OCT-A (superficial capillary plexus) at the superonasal field of Birdshot chorioretinitis with different activity outcomes.

Superonasal Field OCTA	ACTIVE-INACTIVE		*p*-value	ACTIVE-ACTIVE		*p*-value	INACTIVE-INACTIVE		*p*-value
	n	%		n	%		n	%	
**Eyes**	16	26.70		17	28.30		27	45.00	
**Vascular Density (VD) mm-²**	**mean**	**±SD**		**mean**	**±SD**		**mean**	**±SD**	
BL Total VD	8.50	2.96	0.965	9.08	5.60	0.524	8.03	4.99	0.538
12 m Total VD	8.51	4.10	0.283	7.24	5.30	0.832	6.93	4.36	0.445
Δ 12 m - BL	*0.01*	*4.83*	*0.274*	*-1.85*	*3.98*	*0.357*	*-1.10*	*4.14*	*0.890*
BL Central VD	7.68	4.32	0.696	7.86	6.26	0.566	6.52	5.56	0.386
12 m Central VD	7.74	5.43	0.314	6.26	5.51	0.757	6.14	4.88	0.539
Δ 12 m - BL	*0.07*	*6.27*	*0.600*	*-1.60*	*5.39*	*0.423*	*-0.39*	*6.07*	*0.796*
BL Internal VD	8.38	3.65	0.845	8.66	5.69	0.636	7.73	5.36	0.547
12 m Internal VD	8.40	4.63	0.223	6.90	5.40	0.827	6.50	4.48	0.376
Δ 12 m - BL	*0.02*	*5.55*	*0.311*	*-1.76*	*4.01*	*0.476*	*-1.23*	*4.81*	*0.798*
BL External VD	8.57	2.80	0.975	9.27	5.56	0.492	8.20	4.97	0.551
12 m External VD	8.58	3.90	0.323	7.54	5.11	0.940	7.09	4.35	0.417
Δ 12 m - BL	*0.01*	*4.63*	*0.276*	*-1.73*	*4.00*	*0.398*	*-1.11*	*3.99*	*0.840*
**Perfusion Index (PI) %**	**mean**	**±SD**		**mean**	**±SD**		**mean**	**±SD**	
BL Total PI	20.50	7.56	0.970	22.43	14.15	0.462	19.50	12.57	0.527
12 m Total PI	20.73	10.50	0.307	18.23	13.29	0.972	16.56	11.03	0.347
Δ 12 m - BL	*0.22*	*12.53*	*0.258*	*-4.20*	*10.04*	*0.443*	*-2.94*	*10.24*	*0.757*
BL Central PI	18.06	11.04	0.751	18.89	15.07	0.530	15.50	13.45	0.395
12 m Central PI	18.45	13.48	0.402	15.88	14.15	0.951	14.73	12.31	0.490
Δ 12 m - BL	*0.39*	*16.05*	*0.646*	*-3.01*	*13.69*	*0.536*	*-0.77*	*14.87*	*0.879*
BL Internal PI	20.06	9.14	0.954	21.48	14.15	0.549	18.82	13.43	0.552
12 m Internal PI	20.68	11.85	0.241	17.39	14.22	0.962	15.71	11.29	0.317
Δ 12 m - BL	*0.61*	*14.21*	*0.253*	*-4.09*	*9.91*	*0.500*	*-3.11*	*11.83*	*0.684*
BL External PI	20.71	7.18	0.925	22.88	14.19	0.436	19.89	12.51	0.534
12 m External PI	20.82	10.05	0.336	18.59	13.13	0.947	16.91	10.98	0.360
Δ 12 m - BL	*0.11*	*12.13*	*0.257*	*-4.29*	*10.26*	*0.427*	*-2.97*	*9.85*	*0.773*

The *p*-value indicates the deviation from the entire population in the characterization of the A-I, A-A, and I-I groups with the respective characterizing variables. Lighter shades of grey signify a decrease, while darker shades denote an increase from baseline to 12 months of follow-up. $p < 0.05$ is marked in bold. Abbreviations: Optical coherence tomography angiography; BL: baseline; m: months.
