# Supplementary material for: Microvascular and Structural Characterization of Birdshot Chorioretinitis in Active and Inactive Phases
Source: Biomedicines. 2024 Oct 21;12(10):2414. doi: 10.3390/biomedicines12102414 (PMC11505013; doi:10.3390/biomedicines12102414)
Supplement: Supplementary file 1 [file biomedicines-12-02414-s001.zip › Table S4.pdf]

Table S4. Ultra-WideField® spots' features characterize Birdshot chorioretinitis eyes with different activity outcomes.

	ACTIVE-INACTIVE		*p*-value	ACTIVE-ACTIVE		*p*-value	INACTIVE-INACTIVE		*p*-value
	**n**	**%**		**n**	**%**		**n**	**%**	
**Eyes**	16	26.7		17	28.3		27	45.0	
**Spot Size**	**n**	**%**		**n**	**%**		**n**	**%**	
BL Small	7	43.75	0.508	5	29.41	0.487	10	37.04	0.957
12 m Small	7	43.75	0.508	5	29.41	0.487	10	37.04	0.957
Δ 12 m - BL	*0*	*0.00*	*0.378*	*0*	*0.00*	*0.555*	*0*	*0.00*	*0.722*
BL Medium	6	37.50	0.365	7	41.18	0.189	4	14.82	**0.040**
12 m Medium	5	31.25	0.306	4	23.53	0.818	4	14.82	0.266
Δ 12 m - BL	*-1*	*-6.25*	*0.534*	*-3*	*-17.65*	*0.510*	*0*	*0.00*	*0.722*
BL Confluent	3	18.75	0.122	5	29.41	0.592	13	48.15	0.062
12 m Confluent	4	25.00	0.126	8	47.06	0.607	13	48.15	0.373
Δ 12 m - BL	*1*	*6.25*	*0.629*	*3*	*17.65*	*0.265*	*0*	*0.00*	*0.722*
**Spot Location**	**n**	**%**		**n**	**%**		**n**	**%**	
BL Juxtapapillary	6	37.50	0.462	2	11.77	0.056	10	37.04	0.301
12 m Juxtapapillary	6	37.50	0.462	3	17.65	0.206	9	33.33	0.623
Δ 12 m - BL	*0*	*0.00*	*0.516*	*1*	*5.88*	*0.361*	*-1*	*-3.71*	*0.298*
BL Equator	3	18.75	0.534	7	41.88	0.089	5	18.52	0.316
12 m Equator	3	18.75	0.648	6	35.29	0.196	5	18.52	0.448
Δ 12 m - BL	*0*	*0.00*	*0.516*	*-1*	*-6.59*	*0.567*	*0*	*0.00*	*0.842*
BL Diffuse	7	43.75	0.914	8	47.06	0.844	12	44.44	0.940
12 m Diffuse	7	43.75	0.796	8	47.06	0.969	13	48.15	0.840
Δ 12 m - BL	*0*	*0.00*	*0.516*	*0*	*0.00*	*0.737*	*1*	*3.71*	*0.509*
**Pigmentation**	**n**	**%**		**n**	**%**		**n**	**%**	
BL Absent	12	75.00	0.982	14	82.35	0.439	19	70.37	0.473
12 m Absent	12	75.00	0.884	14	82.35	0.347	18	66.67	0.311
Δ 12 m - BL	*0*	*0*	*0.733*	*0*	*0*	*0.717*	*-1*	*-4*	*0.450*
BL Moderate	4	25.00	0.571	3	17.65	0.808	5	18.52	0.810
12 m Moderate	4	25.00	0.705	3	17.65	0.670	6	22.22	0.924
Δ 12 m - BL	*0*	*0*	*0.733*	*0*	*0*	*0.717*	*1*	*4*	*0.450*
BL Severe	0	0.00	0.387	0	0.00	0.361	3	11.11	0.085
12 m Severe	0	0.00	0.387	0	0.00	0.361	3	11.11	0.085
Δ 12 m - BL	*0*	*0*	*0.733*	*0*	*0*	*0.717*	*0*	*0*	*0.450*

The *p*-value indicates the deviation from the entire population in the characterization of the A-I, A-A, and I-I groups with the respective characterizing variables. Lighter shades of grey signify a decrease, while darker shades denote an increase from baseline to 12 months of follow-up. $p < 0.05$ is marked in bold. Abbreviations: UWF: ultra-wide-field pseudocolor retinography; BL: baseline; m: months.
